# Oestrogen binding and risk factors for breast cancer.

**DOI:** 10.1038/bjc.1990.57

**Published:** 1990-02

**Authors:** D. M. Ingram, E. M. Nottage, D. L. Willcox, A. Roberts

**Affiliations:** University Department of Surgery, Queen Elizabeth II Medical Centre, Nedlands, Western Australia.

## Abstract

Although women with breast cancer tend to have a greater proportion of their circulating oestradiol non-protein bound and albumin bound, and less SHBG-bound, than controls, it remains uncertain whether this has an aetiological role or is an effect of the tumour. Oestradiol and its binding to serum proteins was investigated: (a) in relation to risk factors for breast cancer in a normal population; (b) in women with proliferative benign breast disease as a risk group for breast cancer, and women with non-proliferative benign breast disease as a low risk group, as well as breast cancer patients. The strongest associations were with body mass index; the greater the body mass the greater the bioavailability of oestradiol. Changes in relation to age at menarche and menopause could have been a function of body mass. An interesting change with age was noted with a fall in bioavailability over the menopausal years. There was no relationship apparent for parity, age at first full term pregnancy, family history or country of birth. Similar differences in oestradiol binding between cases and controls were seen for patients with breast cancer, benign epithelial hyperplasia and fibrocystic disease without proliferative changes, but these were not significant. This study provides limited support for the concept that oestradiol binding has an aetiological role in the development of breast cancer.


Br. J. Cancer (1990), 61, 303-307 © Macmillan Press Ltd., 1990

### Oestrogen binding and risk factors for breast cancer

D.M. Ingram[1], E.M. Nottage[1], D.L. Willcox[3] & A. Roberts[2]

[1]*University Department of Surgery, and* [2]*State Health Laboratory Services, Queen Elizabeth II Medical Centre, Nedlands 6009, Western Australia; and* [3]*Saren Australia, Science and Biomedical Technology, Australia.*

**Summary** Although women with breast cancer tend to have a greater proportion of their circulating oestradiol non-protein bound and albumin bound, and less SHBG-bound, than controls, it remains uncertain whether this has an aetiological role or is an effect of the tumour. Oestradiol and its binding to serum proteins was investigated: (a) in relation to risk factors for breast cancer in a normal population; (b) in women with proliferative benign breast disease as a risk group for breast cancer, and women with non-proliferative benign breast disease as a low risk group, as well as breast cancer patients. The strongest associations were with body mass index; the greater the body mass the greater the bioavailability of oestradiol. Changes in relation to age at menarche and menopause could have been a function of body mass. An interesting change with age was noted with a fall in bioavailability over the menopausal years. There was no relationship apparent for parity, age at first full term pregnancy, family history or country of birth. Similar differences in oestradiol binding between cases and controls were seen for patients with breast cancer, benign epithelial hyperplasia and fibrocystic disease without proliferative changes, but these were not significant. This study provides limited support for the concept that oestradiol binding has an aetiological role in the development of breast cancer.

For long it has been believed that many forms of breast disease, particularly breast cancer, have a hormonal origin. In particular, because of its profound stimulatory influence on breast ductal epithelium, it was thought that oestradiol must play a central role. Differences in the total concentration of oestradiol, however, are generally not apparent between patients with breast cancer and normal controls, particularly for premenopausal women (Moor *et al.*, 1982; Reed *et al.*, 1983; Ota *et al.*, 1986). In recent years the concept of bioavailability of oestradiol has been recognised, oestradiol being mostly loosely bound to albumin, about one-third tightly bound to sex hormone binding globulin (SHBG) and a few per cent non-protein bound or 'free' (Siiteri *et al.*, 1982). It is the free and albumin bound components which are available to the tissues and are therefore the functionally important proportion of oestradiol. A number of studies have demonstrated that the proportions of free and albumin bound oestradiol are higher in breast cancer patients (Moore *et al.*, 1982; Reed *et al.*, 1983; Ota *et al.*, 1986).

Postulating an aetiological role is difficult as the changes in binding may be an effect of the disease rather than cause. To try to provide further evidence we have investigated associations between known risk factors and oestradiol binding in a study population consisting of breast cancer patients, patients with histologically categorised benign breast disease and 'normal' community controls. The benign breast disease patients were grouped into those who histologically had evidence of epithelial hyperplasia and hence had an increased risk of subsequently developing breast cancer, and those with only changes of fibrosis and cystic disease who should not have had any increase in risk (Page *et al.*, 1978).

### Methods

### *Subjects*

Five hundred and eighteen women were studied between February 1985 and August 1987. Cases were identified from the pathology reports of the combined Hospital and University Pathology Services and the State Health Laboratory Services at the Queen Elizabeth II Medical Centre, Perth, Western Australia. The histology was reviewed by a pathologist (A.R.) who categorised the cases into invasive breast cancer, benign epithelial hyperplasia with or without atypia, or benign fibrocystic disease without any evidence of epithelial proliferation. Patients with other breast pathologies were not included. Each case was matched by age (5-year age group) and area of residence (electoral district) with a control randomly chosen from the electoral roll.

All subjects were contacted by an identical letter requesting their participation in a health survey, but without specific mention of breast disease. Cases were not contacted until consent from their surgeon had been obtained, and not until 3 months had elapsed from the time of their surgery. Failure to respond to the letter was followed by a further letter and telephone call. If a control refused participation a replacement was chosen from the electoral role.

### *Data collection*

All women were interviewed at home by a single interviewer (E.N.). Data relating to risk factors for breast disease were gathered on a previously developed questionnaire, including details of menstrual status, country of birth, oral contraceptive and other hormonal use, family history of breast cancer, age at menarche and menopause, parity and age at first child. Height and weight were measured.

### *Hormone assay*

A single 40 ml fasting blood sample was taken between 8.00 a.m. and 12 midday, the serum was promptly separated and glass vials each containing 1 ml of serum were frozen and stored at −70°C. The specimens were stored at −70°C as oestradiol tends to dissociate with time from its binding proteins when stored at −20°C (Langley *et al.*, 1985). In addition, the duration of storage was similar for cases and controls. Premenopausal women who had not had a hysterectomy had their blood collected on days 21 or 22 of the menstrual cycle. The specimens were assayed in batches for total concentration of oestradiol, progesterone and sex hormone binding globulin (SHBG) by radio-immunoassay using commercial kits, with cases and controls being spread between batches. In addition, women whose menopausal status was uncertain had the concentration of follicle stimulating hormone (FSH) assayed. Menopausal status was thus determined using the concentrations of FSH, oestradiol and progresterone. The coefficients of variation between assays were oestradiol 9%, SHBG 9%, FSH 7% and progesterone 10%. The non-protein bound (free) proportion of oestradiol was determined by rate dialysis (Willcox *et al.*, 1983) and the albumin bound component by the same method after heat treatment of serum at 60°C for 1 h (Hammond *et al.*, 1982).

Correspondence: D.M. Ingram.
Received 28 February 1989; and in revised form 10 October 1989.

### *Statistical methods*

The data were loaded into the data base of a personal computer and statistical analyses performed using the program Epilog (Epicentre Software, Pasadena, CA, USA). Estimates of relative risk and associated 95% confidence limits were determined by conditional logistic regression for each of the hormonal variables and for each of the disease groups studied, i.e. patients with invasive breast cancer, patients with benign epithelial hyperplasia and patients with benign fibrocystic disease without evidence of epithelial hyperplasia. Each hormonal variable was recoded into approximately equal quartiles and the relative risks were expressed for each of the quartiles in relation to the lowest quartile. As body mass was found to have a profound effect on oestrogen binding, all estimates of relative risk were adjusted for body mass index.

Associations between risk factors for breast cancer and each of the hormonal variables were determined by one-way analysis of variance after categorising the risk factor into relevant sub-groups ($F$ test), utilising one-sided $P$ values. In addition, associations between risk factors and the hormonal variables were tested by linear regression analysis and after adjusting for Quetélet's index. Analyses were repeated after logarithmic transformation of the hormonal variable. These associations were determined using only the control population.

Women taking the oral contraceptive pill (25), oestrogen replacement therapy (19), tamoxifen (15) or undergoing cytotoxic therapy (1) were excluded from analyses. In addition, the group of premenopausal women who had had a hysterectomy were excluded from analyses involving total concentration of oestradiol or progesterone as their stage of the menstrual cycle could not be determined (66).

### Results

### *Patients and controls*

One hundred and eight patients with invasive breast cancer, 96 patients with benign epithelial hyperplasia of the breast and 96 patients with fibrocystic disease of the breast but without epithelial hyperplasia were studied. The patients with epithelial hyperplasia and fibrocystic disease shared a common control subject, and in total 214 community control subjects were studied. Seventy-eight per cent of contactable control subjects agreed to take part in the study while 84% of contacted patients took part.

### *Age*

Both variables of bioavailability of oestradiol, i.e. the free and albumin bound components, showed the same pattern of being high in the young age group and progressively falling, reaching a low in the perimenopausal years, thereafter rising as age increased. This pattern was supported by the changes in SHBG concentration which were diametrically opposite, reaching a peak in the perimenopausal years (Figure 1 and Table I). Adjusting for QI improved the statistical significance for these associations (free oestradiol $P = 0.0156$, albumin-bound oestradiol $P = 0.0116$, SHBG $P = 0.5297$).

### *Country of birth*

There were few Europe-born and Asia-born women. There was little difference between the other countries of birth for any of the hormonal variables. This applied even after re-analysis for those who had been in Australia for less than 15 years (Table I).

### *Age at menarche*

The recalled age at menarche was plotted for each of the hormonal variables (Figure 2). The only pattern to emerge was a stepwise fall in SHBG concentration for decreasing recalled age at menarche. This was not statistically significant (Table I). Similarly, when analysed by linear regression there were no significant associations with the hormonal variables.

**Figure 1** The proportion of free and albumin bound oestradiol, and concentration of SHBG plotted by age in 10-year age groups for normal population, showing a fall in oestradiol binding and a rise in SHBG in the perimenopausal years. These changes appear to be independant of body mass changes. (Mean ± s.e.m.)

### *Age at menopause*

The recalled age at menopause was plotted for each of the hormonal variables (Figure 2). For both the total concentration of oestradiol and the proportion of oestradiol which was bioavailable (free and albumin bound) there was a progressive stepwise increase with increasing age at menopause. Similarly, for the concentration of SHBG there was a reduction in SHBG with increasing age at menopause (Table I). After adjusting for QI, however, these patterns largely disappeared, as did any trend to statistical significance (free oestradiol $P = 0.8943$, albumin bound oestradiol $P = 0.8458$, SHBG $P = 0.8058$). Linear regression analysis was not significant for any of the hormonal variables.

**Figure 2** The proportion of free and albumin-bound oestradiol, and concentration of SHBG plotted against the recalled age at menarche (**a**) and age at menopause (**b**) for the population of normal women. The apparent rise in oestradiol binding and fall in SHBG with increasing age at menopause largely disappeared after adjusting for body mass. (Mean ± s.e.m.)

**Table I** Oestrogen variable (mean ± s.e.m.)

| Risk variable | No. | Total oestradiol: Premenopausal (pmol $l^{-1}$) | Total oestradiol: Post-menopausal (pmol $l^{-1}$) | 'Free' oestradiol (%) | Albumin bound oestradiol (%) | SHBG (nmol $l^{-1}$) |
|---|---|---|---|---|---|---|
| Age (years) | | | | | | |
| 30 or less | 7 | 376 ± 75 | – | 1.40 ± 0.10 | 69.3 ± 6.2 | 63.7 ± 6.7 |
| 31–40 | 30 | 362 ± 35 | – | 1.37 ± 0.05 | 64.4 ± 2.4 | 71.2 ± 5.1 |
| 41–50 | 69 | 432 ± 47 | 21.1 ± 4.6 | 1.27 ± 0.03 | 60.9 ± 1.2 | 77.2 ± 5.7 |
| 51–60 | 32 | – | 33.6 ± 6.0 | 1.30 ± 0.04 | 63.7 ± 2.2 | 69.9 ± 7.8 |
| 61 or more | 51 | – | 26.1 ± 4.8 | 1.33 ± 0.04 | 67.5 ± 2.0 | 65.1 ± 6.3 |
| | | | | $P = 0.255$ | $P = 0.047$ | $P = 0.625$ |
| Country of birth | | | | | | |
| NZ | 13 | 558 ± 191 | 37.2 ± 12.6 | 1.30 ± 0.08 | 64.2 ± 3.4 | 69.1 ± 13.6 |
| UK | 48 | 329 ± 37 | 35.0 ± 6.6 | 1.31 ± 0.03 | 62.6 ± 1.7 | 70.8 ± 5.8 |
| Europe | 7 | 255 ± 55 | 10.0 ± 5.0 | 1.35 ± 0.09 | 63.9 ± 4.5 | 62.3 ± 15.2 |
| Asia | 7 | – | 12.4 ± 5.2 | 1.22 ± 0.08 | 64.0 ± 4.5 | 68.2 ± 11.6 |
| Australia | 108 | 449 ± 50 | 20.7 ± 3.0 | 1.31 ± 0.02 | 64.9 ± 1.3 | 70.9 ± 4.2 |
| Age at menarche (years) | | | | | | |
| 11 or less | 23 | 398 ± 96 | 19.7 ± 6.4 | 1.30 ± 0.05 | 65.3 ± 2.6 | 66.1 ± 8.6 |
| 12–13 | 83 | 383 ± 27 | 24.3 ± 4.5 | 1.32 ± 0.03 | 65.0 ± 1.5 | 68.7 ± 4.6 |
| 14–15 | 65 | 479 ± 107 | 29.5 ± 5.6 | 1.29 ± 0.03 | 62.0 ± 1.3 | 74.8 ± 5.0 |
| 16 or more | 16 | 391 ± 52 | 21.2 ± 6.2 | 1.31 ± 0.05 | 64.8 ± 3.0 | 75.7 ± 14.1 |
| | | $P = 0.715$ | $P = 0.668$ | $P = 0.894$ | $P = 0.483$ | $P = 0.745$ |
| Age at menopause (years) | | | | | | |
| 40 or less | 17 | – | 19.5 ± 4.6 | 1.20 ± 0.06 | 59.8 ± 2.7 | 77.3 ± 11.0 |
| 41–45 | 16 | – | 18.8 ± 4.3 | 1.22 ± 0.06 | 65.1 ± 3.4 | 70.3 ± 8.5 |
| 46–50 | 29 | – | 24.6 ± 4.4 | 1.34 ± 0.04 | 66.1 ± 1.9 | 57.9 ± 6.0 |
| 51 or more | 19 | – | 34.1 ± 8.8 | 1.35 ± 0.05 | 68.7 ± 3.4 | 62.1 ± 12.4 |
| | | | $P = 0.277$ | $P = 0.081$ | $P = 0.179$ | $P = 0.439$ |
| Quetélet's index (kg $m^{-2}$) | | | | | | |
| 20 or less | 16 | 538 ± 113 | 19.2 ± 4.8 | 1.22 ± 0.05 | 59.4 ± 3.2 | 89.2 ± 12.2 |
| 21–24 | 71 | 356 ± 36 | 24.0 ± 4.1 | 1.22 ± 0.03 | 59.0 ± 1.5 | 82.5 ± 5.2 |
| 25–28 | 57 | 461 ± 98 | 26.0 ± 5.6 | 1.32 ± 0.03 | 65.5 ± 1.5 | 68.0 ± 4.8 |
| 29 or more | 43 | 374 ± 47 | 28.4 ± 6.6 | 1.47 ± 0.03 | 71.9 ± 1.5 | 49.5 ± 5.8 |
| | | $P = 0.348$ | $P = 0.884$ | $P = 0.0001$ | $P = 0.0001$ | $P = 0.0002$ |
| Parity (no. children) | | | | | | |
| 0 | 22 | 432 ± 89 | 13.3 ± 13.4 | 1.27 ± 0.06 | 64.2 ± 3.1 | 69.3 ± 6.3 |
| 1 | 22 | 326 ± 55 | 33.5 ± 10.6 | 1.35 ± 0.04 | 66.5 ± 2.4 | 60.8 ± 8.6 |
| 2 | 46 | 289 ± 41 | 33.9 ± 7.3 | 1.26 ± 0.03 | 61.9 ± 1.6 | 76.4 ± 6.1 |
| 3 | 55 | 454 ± 51 | 22.2 ± 5.9 | 1.33 ± 0.04 | 65.3 ± 1.9 | 75.9 ± 6.8 |
| 4 or more | 44 | 512 ± 120 | 22.2 ± 3.9 | 1.32 ± 0.03 | 63.1 ± 1.8 | 66.3 ± 3.1 |
| | | $P = 0.219$ | $P = 0.169$ | $P = 0.402$ | $P = 0.547$ | $P = 0.516$ |
| Age at first pregnancy (years) | | | | | | |
| 20 or less | 25 | 254 ± 35 | 19.9 ± 5.5 | 1.25 ± 0.05 | 61.1 ± 2.5 | 77.1 ± 8.7 |
| 21–24 | 65 | 502 ± 67 | 25.6 ± 3.7 | 1.32 ± 0.03 | 63.7 ± 1.5 | 74.3 ± 5.5 |
| 25–28 | 36 | 436 ± 97 | 29.3 ± 8.2 | 1.29 ± 0.04 | 63.9 ± 2.2 | 69.0 ± 6.6 |
| 29 or more | 23 | 251 ± 70 | 33.8 ± 10.1 | 1.34 ± 0.04 | 65.2 ± 2.9 | 69.9 ± 10.5 |
| | | $P = 0.104$ | $P = 0.684$ | $P = 0.538$ | $P = 0.701$ | $P = 0.877$ |
| Family history | | | | | | |
| No FH | 164 | (insufficient numbers for | | 1.31 ± 0.02 | 64.5 ± 1.0 | 71.9 ± 3.4 |
| 2nd degree | 12 | separate analyses) | | 1.24 ± 0.05 | 58.2 ± 2.0 | 71.6 ± 8.3 |
| 1st degree | 12 | – | – | 1.32 ± 0.06 | 62.9 ± 3.2 | 61.7 ± 10.3 |

$P$ = statistical significance based on unadjusted determination by analysis of variance.

*Body mass index*

Body mass index as determined by Quetélet's index (kg $m^{-2}$) was highly significantly related to the binding of oestradiol. With increasing obesity there was a progressive rise in the proportion of free and albumin bound oestradiol, and a progressive reduction in the SHBG concentration (Figure 3). For post-menopausal women there was a progressive rise in total oestradiol with increasing obesity, but this did not apply for premenopausal women (Table I). Linear regression analysis confirmed these associations.

*Parity*

There were no associations apparent between the variables of oestradiol binding and the number of full term pregnancies.

*Age at first pregnancy*

As with parity there were no associations between oestradiol binding and the age at first pregnancy although again there

**Figure 3** Body mass and oestradiol binding. The proportion of free and albumin-bound oestradiol plotted against body mass (Quetélet's index). Strong associations are apparent. (Mean ± s.e.m.)

did appear to be an association with the total concentration of oestradiol for post-menopausal women. Post-menopausal women who had a late age at first pregnancy had a higher concentration of total oestradiol but this again was not significant (Table I).

*Family history*

There did not appear to be any association between family history of breast cancer and oestradiol binding although women who had a first degree relative who had had breast cancer had a rather lower SHBG concentration. This was not statistically significant. The number of women in the study group with a family history of breast cancer was relatively small and when divided into sub-groups, e.g. pre and post-menopausal or multiple relatives, the numbers were too small to be meaningful (Table I).

*Benign breast disease*

The relative risks of cases in relation to controls were determined for each of the hormonal variables for breast cancer patients, for patients with benign epithelial hyperplasia and for patients with fibrocystic disease of the breast without evidence of proliferative changes (Table II). Because of the influence of obesity and age on oestrogen binding, all estimates of relative risk were adjusted for Quetélet's index and age and the lines of regression for each hormonal variable against Quetélet's index were plotted separately for cases and controls (Figure 4). Similar patterns were apparent for the cancer patients as for the patients with benign epithelial hyperplasia and those with fibrocystic disease. In each situation the cases had a higher level of free and albumin bound oestradiol at all levels of obesity compared to their controls, and conversely had lower concentrations of SHBG at all levels of obesity compared to controls. In none of these situations, however, was statistical significance reached.

### Discussion

One of the problems with case–control studies is knowing if differences between patients and their controls relate to the cause of the disease or occur because of the disease process. As regards the role of oestradiol and its binding to serum

**Figure 4** Comparison of oestradiol binding for patients and controls at differing levels of obesity. The lines of regression for patients (heavy lines) and controls (fine lines) plotted for each of the disease states studied: breast cancer, benign epithelial hyperplasia and fibrocystic disease of the breast. At all levels of body mass cases had a greater proportion of their oestradiol free and albumin body and a lower SHBG concentration compared to controls. **a**, breast cancer; **b**, benign epithelial hyperplasia; **c**, fibrocystic disease of the breast.

proteins in the aetiology of carcinoma of the breast, we have attempted to resolve the problem by: (a) looking at differences in these hormonal variables for differing levels of risk in a control population; (b) undertaking a series of case–control studies, not only for patients with breast cancer but also for patients with histologically proven benign epithelial hyperplasia who are thus at risk of developing breast cancer, and also for a low risk group, patients with fibrocystic disease but with no proliferative changes on histology (Page *et al.*, 1978). If the trends seen in the breast cancer patients were also reflected in the high risk benign breast disease group, then this would be further evidence that oestradiol binding plays an aetiological role.

From the data presented in this paper, although a number of trends emerge, there is little conclusive evidence of associations between oestradiol and its binding and risk factors for breast cancer. Of all the variables of risk studied, body mass had by far the strongest association (de Moor & Joossens, 1970) and may in fact, as discussed below, be responsible for many of the trends seen with the other risk factors. We have demonstrated that women with a large body mass have a much greater proportion of their oestradiol bioavailable, i.e. free or albumin bound. Obesity itself is not a strong risk factor for breast cancer, de Waard *et al.* (1974) finding that only women over 65 who weighed more than 80 kg had an increased risk for breast cancer, while our own studies have shown that women who gain more than 10 kg over their reproductive years have an increased relative risk which is approximately two-fold (Ingram *et al.*, 1989). It should be noted that any risk, however small, if it is widely prevalent in the study population (as is obesity), can have a major impact on the incidence of the disease.

**Table II** Estimations of relative risks for each of the disease study groups in relation to controls, after adjusting for age and Quetélet's index.

| | *Breast cancer* | *Benign epithelial hyperplasia* | *Fibrocystic disease of the breast* |
|---|---|---|---|
| *Free oestradiol* | | | |
| 1st quartile | 1.0 | 1.0 | 1.0 |
| 2nd quartile | 2.2 | 3.0 | 2.2 |
| 3rd quartile | 2.0 | 2.3 | 2.9 |
| 4th quartile | 0.8 | 2.3 | 2.2 |
| $\chi^2$ (3 d.f.) | 6.10 | 4.74 | 5.02 |
| *Albumin bound oestradiol* | | | |
| 1st quartile | 1.0 | 1.0 | 1.0 |
| 2nd quartile | 1.4 | 1.3 | 2.0 |
| 3rd quartile | 1.1 | 1.4 | 1.6 |
| 4th quartile | 1.4 | 2.3 | 2.0 |
| $\chi^2$ (3 d.f.) | 0.64 | 3.08 | 2.20 |
| *SHBG concentration* | | | |
| 1st quartile | 1.0 | 1.0 | 1.0 |
| 2nd quartile | 2.0 | 1.1 | 1.6 |
| 3rd quartile | 1.5 | 0.6 | 0.4 |
| 4th quartile | 2.0 | 0.9 | 0.7 |
| $\chi^2$ (3 d.f.) | 2.42 | 2.16 | 7.22 |

The estimates have been undertaken for the proportion of free oestradiol, the proportion bound to albumin, and for the concentration of SHBG. Statistical significance was not reached in any situation.

The association of oestradiol binding with age is interesting if one considers the relationship between breast cancer incidence and age in Western population. There is a steep rise to age 40 and thereafter the incidence plateaus until the post-menopausal years when it rises again (Fleming *et al.*, 1981). This fits very nicely with the changes in oestradiol binding demonstrated in Figure 1, where the proportion of bioavailable oestradiol is high in the premenopausal years, falls over the menopausal years and is high again in later life. SHBG follws a converse pattern. One possibility is that the post-menopausal rise in free and albumin bound oestradiol (and fall in SHBG) occurs because of weight gain in these years, but adjusting for body mass index appeared to strengthen rather than reduce these associations.

The patterns of rising free and albumin bound oestradiol and falling SHBG with increase in age at menopause, and fall in SHBG with early age at menarche, support these hormonal changes as having a role in breast cancer development as early age at menarche and late age at menopause are well recognised risk factors (Pike *et al.*, 1981). Again, however, these changes could be accounted for by obesity as we have demonstrated in a previous study that obese women are more likely to have an early age of menarche and late age at menopause (Ingram *et al.*, 1989). Moore *et al.* (1987) similarly demonstrated that late menarche was associated with increased SHBG and adjusting for QI reduced the magnitude of the association; we have demonstrated here that adjusting for body mass reduced the assocation between age at menopause and oestradiol binding and SHBG. Little can be made of the other variables of risk and their associations with oestradiol and its binding. With the country of birth, even after taking out those who have been in Australia for more than 15 years, there were no significant differences although the numbers were small for all other than United Kingdom immigrants. Parity and age at first full term pregnancy were not significantly associated with the hormonal variables while the numbers of women in the control group with a family history of breast cancer were small and it would require a much larger study to evaluate this variable.

As regards differences in oestradiol binding between cases with breast cancer, benign epithelial hyperplasia or fibrocystic disease and their respective controls, there is a remarkably similar pattern throughout, in that after adjusting for body mass, for each group the free and albumin bound proportions of oestradiol are higher for the breast disease patients than controls, and the SHBG concentrations are correspondingly lower (Figure 4). This suggests that increased bioavailability of oestradiol may promote breast cancer by stimulating epithelial growth, although while the estimations of relative risk were correspondingly increased (for free and albumin-bound oestradiol) and lower (for SHBG), in no case did they reach statistical significance.

In conclusion, apart from the association between oestradiol binding and body mass, we have been unable to provide strong evidence that the degree of oestradiol binding to serum proteins is associated with risk factors for the development of breast cancer.

We would like to thank the Cancer Foundation of Western Australia, the Sir Charles Gairdner Hospital Research Foundation and the various Western Australian women's support groups for their financial assistance with this project. In addition we would like to thank Mr Frank Watson of the Department of Clinical Biochemistry for assistance with assays, Dr Dallas English for his guidance with statistical methods, Mrs Peta Diffen for help with collating the data, the Western Australian surgeons who kindly allowed their patients to be studied and the women who unselfishly gave up their time to participate in the study.

### References

DE MOOR, P. & JOOSSENS, J.V. (1970). An inverse relation between body weight and the activity of the steroid binding beta-globulin in human plasma. *Steroidologia*, **1**, 129.

DE WAARD, F. & BAANDERS-VAN HALEWIJN, E.A. (1974). A prospective study in general practice on breast-cancer risk in post-menopausal women. *Int. J. Cancer*, **14**, 153.

FLEMING, N.T., ARMSTRONG, B.K., SHEINER, H.J. & JAMES, I.R. (1981). The occurrence of breast cancer in Australian women. *Med. J. Aust.*, **i**, 289.

HAMMOND, G.L., LAHTEENMAKI, P.L.A., LAHTEENMAKI, P. & LUUKKAINEN, T. (1982). Distribution and percentages of non-protein-bound contraceptive steroids in human serum. *J. Steroid Biochem.*, **17**, 375.

INGRAM, D.M., NOTTAGE, E., NG, S., SPARROW, L., ROBERTS, A. & WILLCOX, D. (1989). Obesity and breast disease: the role of female sex hormones. *Cancer*, **64**, 1049.

LANGLEY, M.S., HAMMOND, G.L., BARDSLEY, A., SELLWOOD, R.A. & ANDERSON, D.C. (1985). Serum steroid binding proteins and the bioavailability of estradiol in relation to breast diseases. *J. Natl Cancer Inst.*, **75**, 823.

MOORE, J.W., CLARK, G.M.G., BULBROOK, R.D., MURAI, J.T., HAMMOND, G.L. & SIITERI, P. (1982). Serum concentrations of total and non-protein-bound oestradiol in patients with breast cancer and in normal controls. *Int. J. Cancer*, **29**, 17.

MOORE, J.W., KEY, T.J.A., BULBROOK, R.D. & 4 others (1987). Sex hormone binding globulin and risk factors for breast cancer in a population of normal women who had never used exogenous sex hormones. *Br. J. Cancer*, **56**, 661.

OTA, D.M., JONES, L.A., JACKSON, G.L., JACKSON, P.M., KAMP, K. & BAUMAN, D. (1986). Obesity, non-protein-bound estradiol levels, and distribution of estradiol in the sera of breast cancer patients. *Cancer*, **57**, 558.

PAGE, D.L., VANDER ZWAAG, R., ROGERS, L.W., WILLIAMS, L.T., WALKER, W.E. & HARTMANN, W.H. (1978). Relation between component parts of fibrocystic disease complex and breast cancer. *J. Natl Cancer Inst.*, **61**, 1055.

PIKE, M.C., HENDERSON, B.E. & CASAGRANDE, J.T. (1981). The epidemiology of breast cancer as it relates to menarche, pregnancy and menopause. *Banbury Report 8: Hormones and Breast Cancer*. Cold Spring Harbor Laboratory: New York.

REED, M.J., CHENG, R.W., NOEL, C.T., DUDLEY, H.A.F. & JAMES, V.H.T. (1983). Plasma levels of estrone, estrone sulfate, and estradiol and the percentage of unbound estradiol in postmenopausal women with and without breast disease. *Cancer Res.*, **43**, 3940.

SIITERI, P.K., MURAI, J.T., HAMMOND, G.L., NISKER, J.A., RAYMOURE, W.J. & KUHN, R.W. (1982). The serum transport of steroid hormones. *Recent Prog. Hormone Res.*, **38**, 457.

WILLCOX, D.L., MCCOLM, S.C., ARTHUR, P.G. & YOVICH, J.L. (1983). The application of rate dialysis to the determination of free steriods in plasma. *Anal. Biochem.*, **135**, 304.


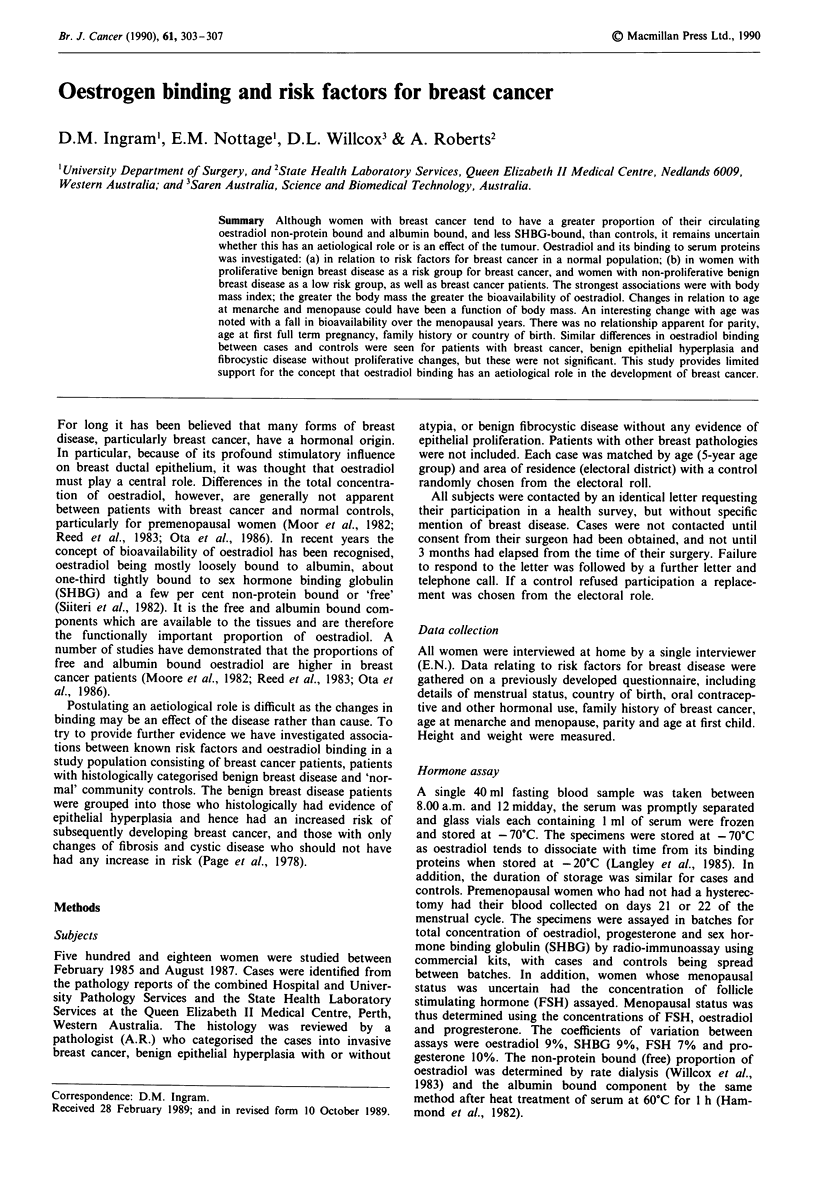

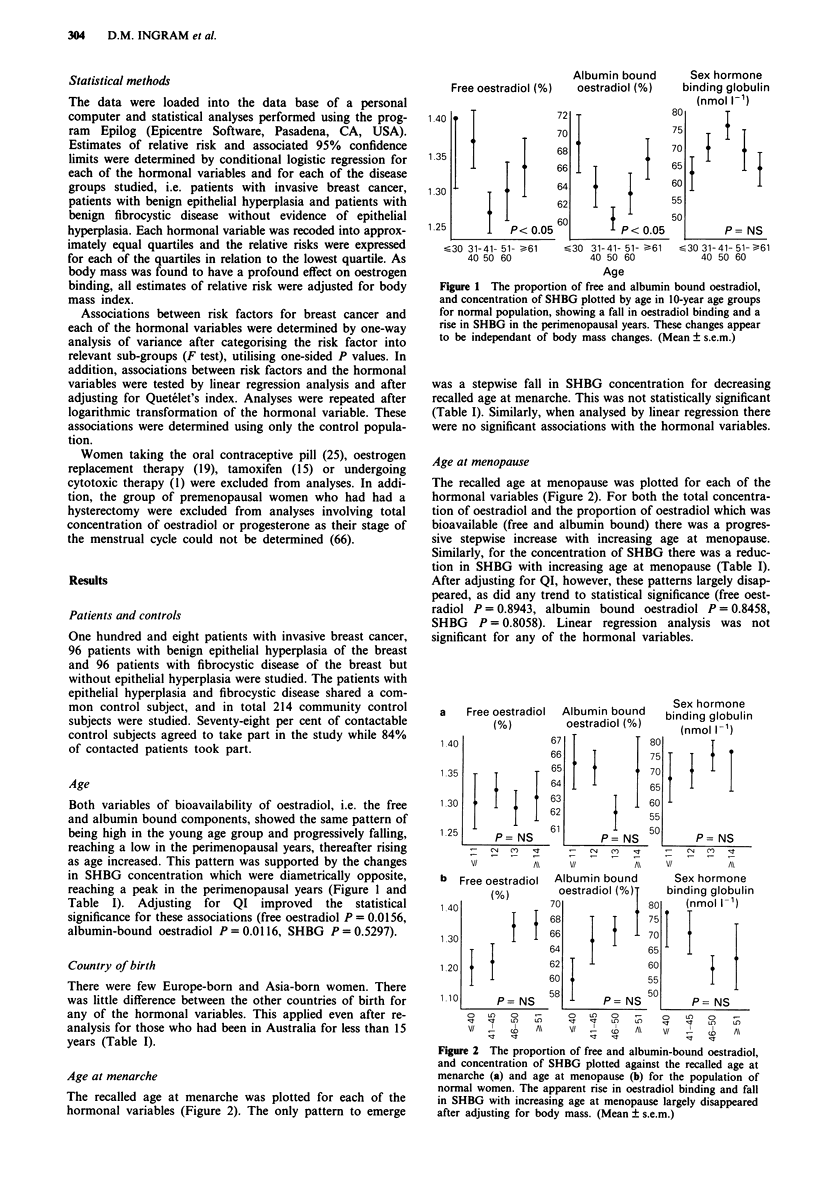

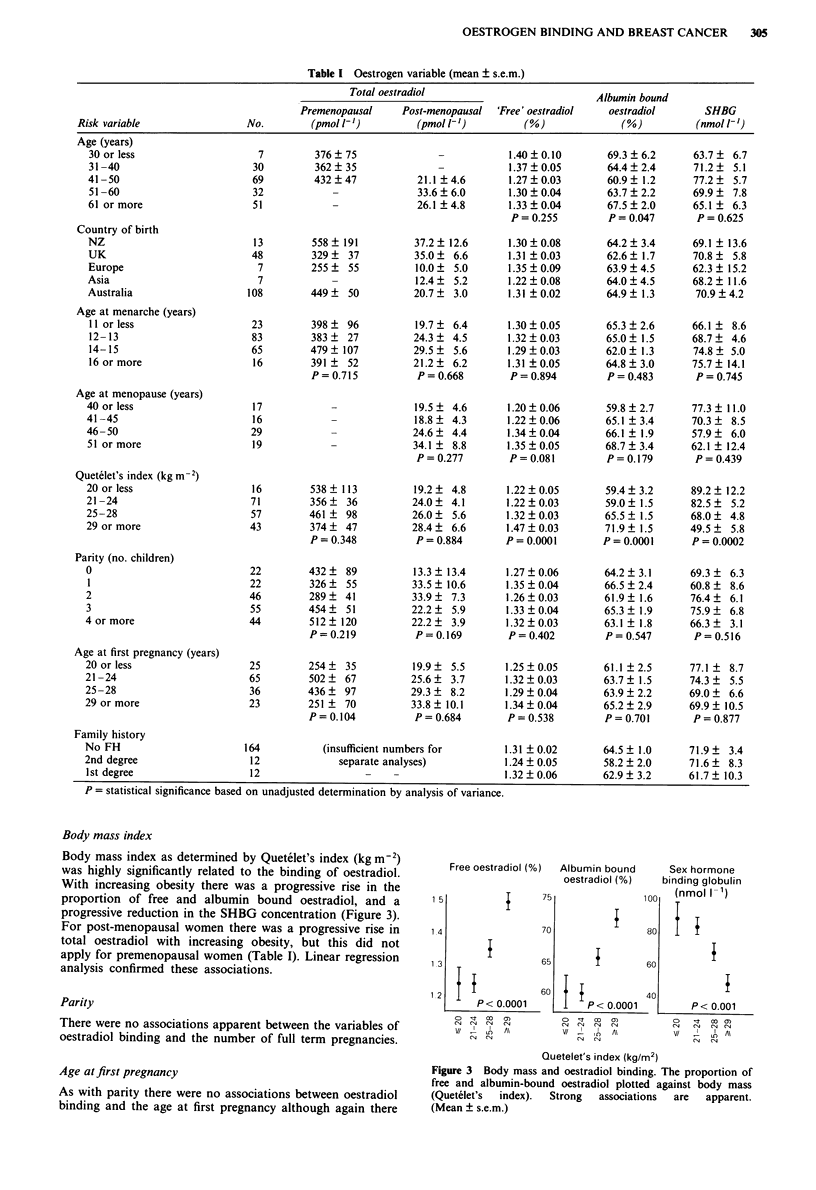

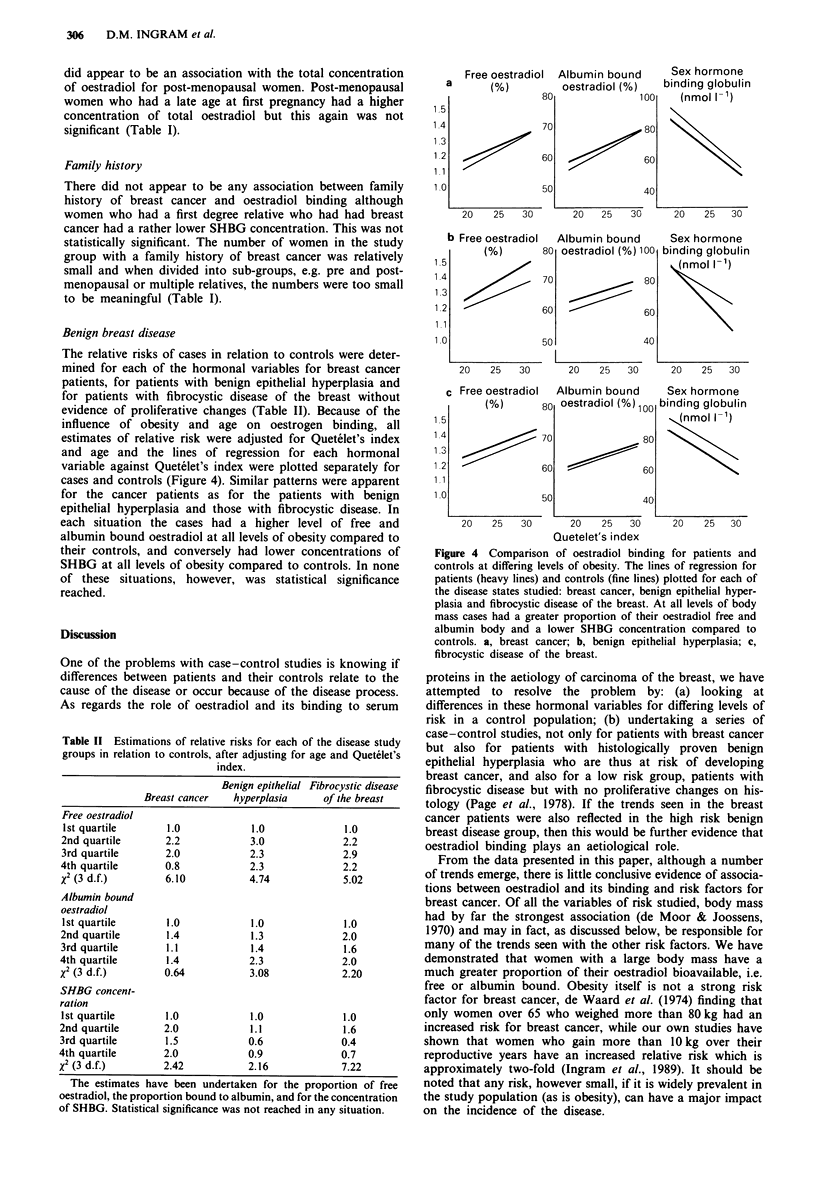

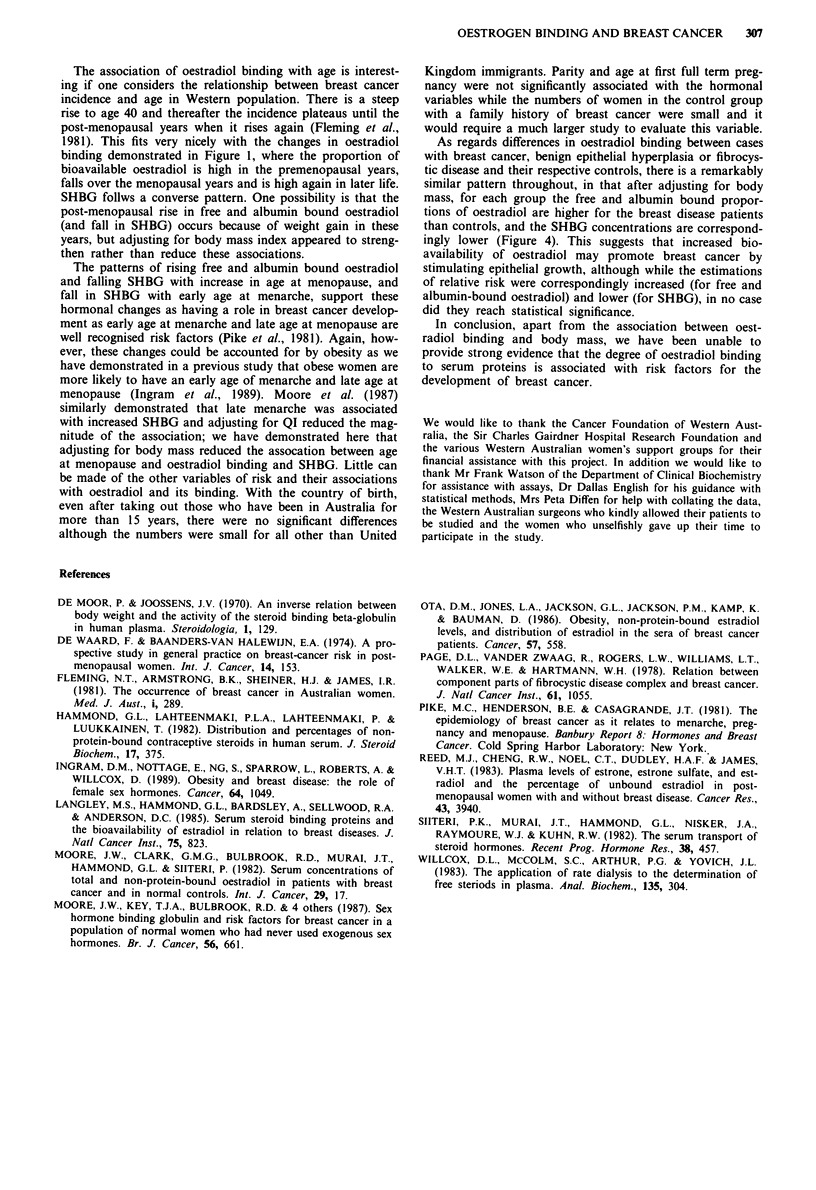


## References

[OCR_01054] Fleming N. T., Armstrong B. K., Sheiner H. J., James I. R. (1981). Occurrence of breast cancer in Australian women.. Med J Aust.

[OCR_01059] Hammond G. L., Lähteenmäki P. L., Lähteenmäki P., Luukkainen T. (1982). Distribution and percentages of non-protein bound contraceptive steroids in human serum.. J Steroid Biochem.

[OCR_01065] Ingram D., Nottage E., Ng S., Sparrow L., Roberts A., Willcox D. (1989). Obesity and breast disease. The role of the female sex hormones.. Cancer.

[OCR_01070] Langley M. S., Hammond G. L., Bardsley A., Sellwood R. A., Anderson D. C. (1985). Serum steroid binding proteins and the bioavailability of estradiol in relation to breast diseases.. J Natl Cancer Inst.

[OCR_01076] Moore J. W., Clark G. M., Bulbrook R. D., Hayward J. L., Murai J. T., Hammond G. L., Siiteri P. K. (1982). Serum concentrations of total and non-protein-bound oestradiol in patients with breast cancer and in normal controls.. Int J Cancer.

[OCR_01082] Moore J. W., Key T. J., Bulbrook R. D., Clark G. M., Allen D. S., Wang D. Y., Pike M. C. (1987). Sex hormone binding globulin and risk factors for breast cancer in a population of normal women who had never used exogenous sex hormones.. Br J Cancer.

[OCR_01088] Ota D. M., Jones L. A., Jackson G. L., Jackson P. M., Kemp K., Bauman D. (1986). Obesity, non-protein-bound estradiol levels, and distribution of estradiol in the sera of breast cancer patients.. Cancer.

[OCR_01096] Page D. L., Vander Zwaag R., Rogers L. W., Williams L. T., Walker W. E., Hartmann W. H. (1978). Relation between component parts of fibrocystic disease complex and breast cancer.. J Natl Cancer Inst.

[OCR_01106] Reed M. J., Cheng R. W., Noel C. T., Dudley H. A., James V. H. (1983). Plasma levels of estrone, estrone sulfate, and estradiol and the percentage of unbound estradiol in postmenopausal women with and without breast disease.. Cancer Res.

[OCR_01113] Siiteri P. K., Murai J. T., Hammond G. L., Nisker J. A., Raymoure W. J., Kuhn R. W. (1982). The serum transport of steroid hormones.. Recent Prog Horm Res.

[OCR_01118] Willcox D. L., McColm S. C., Arthur P. G., Yovich J. L. (1983). The application of rate dialysis to the determination of free steroids in plasma.. Anal Biochem.

[OCR_01044] de Moor P., Joossens J. V. (1970). An inverse relation between body weight and the activity of the steroid binding -globulin in human plasma.. Steroidologia.

[OCR_01049] de Waard F., Baanders-van Halewijn E. A. (1974). A prospective study in general practice on breast-cancer risk in postmenopausal women.. Int J Cancer.

